# Quantification of fractional tumor burden for the early detection of post-treatment glioblastoma progression

**DOI:** 10.1186/s41747-026-00685-3

**Published:** 2026-03-02

**Authors:** Siem Herings, Rebecca de Wit, Baris Saglik, Manoj Mannil, Rik van den Elshout, Anne Arens, Anja van der Kolk, Tom Scheenen, Dylan Henssen

**Affiliations:** 1 Department of Medical Imaging, Radboud university medical center, Nijmegen, The Netherlands; 2 Radboudumc Center of Expertise Neuro-Oncology, Nijmegen, The Netherlands; 3 University Clinic for Radiology, Westfälische Wilhelms-University Muenster and University Hospital Muenster, Muenster, Germany

**Keywords:** Cerebral blood volume, Glioblastoma, Magnetic resonance imaging, Perfusion imaging, Treatment outcome

## Abstract

**Objectives:**

Quantitative postprocessing of perfusion-weighted magnetic resonance imaging, including fractional tumor burden (FTB) maps, provides better visualization of the heterogeneous nature of glioblastomas. This study aimed to determine whether FTB maps help in distinguishing tumor progression (TP) from treatment-related abnormalities (TRA) in post-treatment glioblastoma patients.

**Materials and methods:**

Unenhanced and contrast-enhanced T1-weighted and perfusion-weighted sequences of patients with new contrast-enhancing lesions were retrospectively included. Semiautomatic segmentation of these lesions was performed. Using predefined relative cerebral blood volume (rCBV) thresholds, voxels within this segmentation were classified as FTB_low_, FTB_mid_, or FTB_high._ Patient outcome was determined by clinical and radiological follow-up. Non-parametric statistics were used to compare the FTB quantification. Diagnostic accuracy was evaluated with the area under the receiver operating characteristic curve (AUROC) and Youden’s J. The difference between AUROCs was tested using bootstrapping.

**Results:**

Fifty-nine patients were included, 35 of them showing TP (59%). The percentages of voxels classified as FTB_low_ and FTB_high_ were significantly different between the groups (*p* = 0.031 and *p* = 0.010, respectively). Using the percentage of voxels classified as FTB_high_ as a cutoff to differentiate TP from TRA yielded an AUROC of 0.70 (95% confidence interval: 0.56‒0.84), while FTB_low_ yielded 0.67 (0.52–0.82), without a significant difference (*p* = 0.466). The highest sensitivity and specificity based on the cutoff of 24% of voxels classified as FTB_high_ coverage, were 63% and 79%, respectively.

**Conclusion:**

FTB quantification yielded fair accuracy in the early detection of glioblastoma TP. Future research is needed to investigate how to use FTB maps in clinical practice.

**Relevance statement:**

Early discrimination between TP and TRA, even with fair accuracy, can help in alleviating some uncertainty in glioblastoma patients. A clear visualization of lesion heterogeneity provided by FTB-maps could allow for more targeted treatment options and targeted follow-up.

**Key Points:**

- Follow-up of patients with glioblastoma is complicated by the similar appearance of treatment effects and tumor growth on MRI.
- Perfusion imaging provides a basis for the creation of FTB maps. These visualize the heterogeneity of brain lesions.
- Quantitative analysis of FTB maps can help differentiate tumor growth from treatment effect with reasonable accuracy.

**Graphical Abstract:**

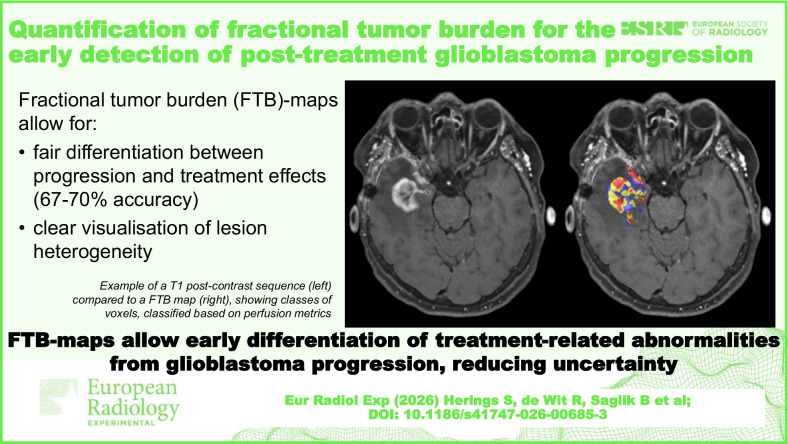

## Background

Glioblastoma is the most common and aggressive glioma subtype in adults [1]. Post-treatment radiological surveillance of glioblastoma is challenged by an unreliable differentiation between tumor progression (TP) and treatment-related abnormalities (TRA) [2–5]. TP concerns the growth of new contrast-enhancing lesions from therapy-resistant glioma cells in the post-treatment setting. TRA, on the other hand, comprises phenomena such as radio necrosis and pseudoprogression. Both present as new contrast-enhancing lesions due to changes in (micro)vascular integrity or cell necrosis after treatment with chemotherapy in combination with radiation therapy [3, 5]. Although much remains elusive regarding the occurrence of TRA in glioblastoma patients who have received concomitant chemoradiation therapy, it is known that TRA occurrence is impacted by the O6-methylguanine-deoxyribonucleic acid methyltransferase (MGMT) promoter status. More specifically, TRA occurs more frequently in patients who show hypermethylation of the MGMT promoter [6, 7].

The differentiation between TP and TRA is relevant as patients presenting with TP could potentially benefit from further treatment, while patients presenting with TRA will not benefit and might worsen due to treatment side effects. Current treatment options are unable to prevent the inevitable recurrence of glioblastoma lesions, and the median survival rate remains abysmal [8]. However, the early identification of TP and the development of new targeted treatment options might change this in the future.

To aid in the early detection of TP, several imaging techniques have been suggested [9–13]. One of these techniques includes perfusion-weighted MRI with a dynamic susceptibility contrast (DSC) sequence. This type of perfusion-weighted imaging visualizes the perfusion of tissue, and its use in glioblastomas is based on the fact that tumor tissue has an increased demand for nutrients, *i.e*., blood, while TRA does not. Despite its extensive usage in clinical practice, the usage of DSC perfusion is still limited by a lack of consensus on acquisition techniques, post-processing methodologies, and interpretation [10].

The reference standard for distinguishing TP from TRA thus remains neurosurgical biopsy, in which accuracy greatly depends on the invasive biopsy site, the sampling of representative tissue, and lesion heterogeneity. A noninvasive alternative for neurosurgical biopsy is preferable [5].

**Fig. 1:**
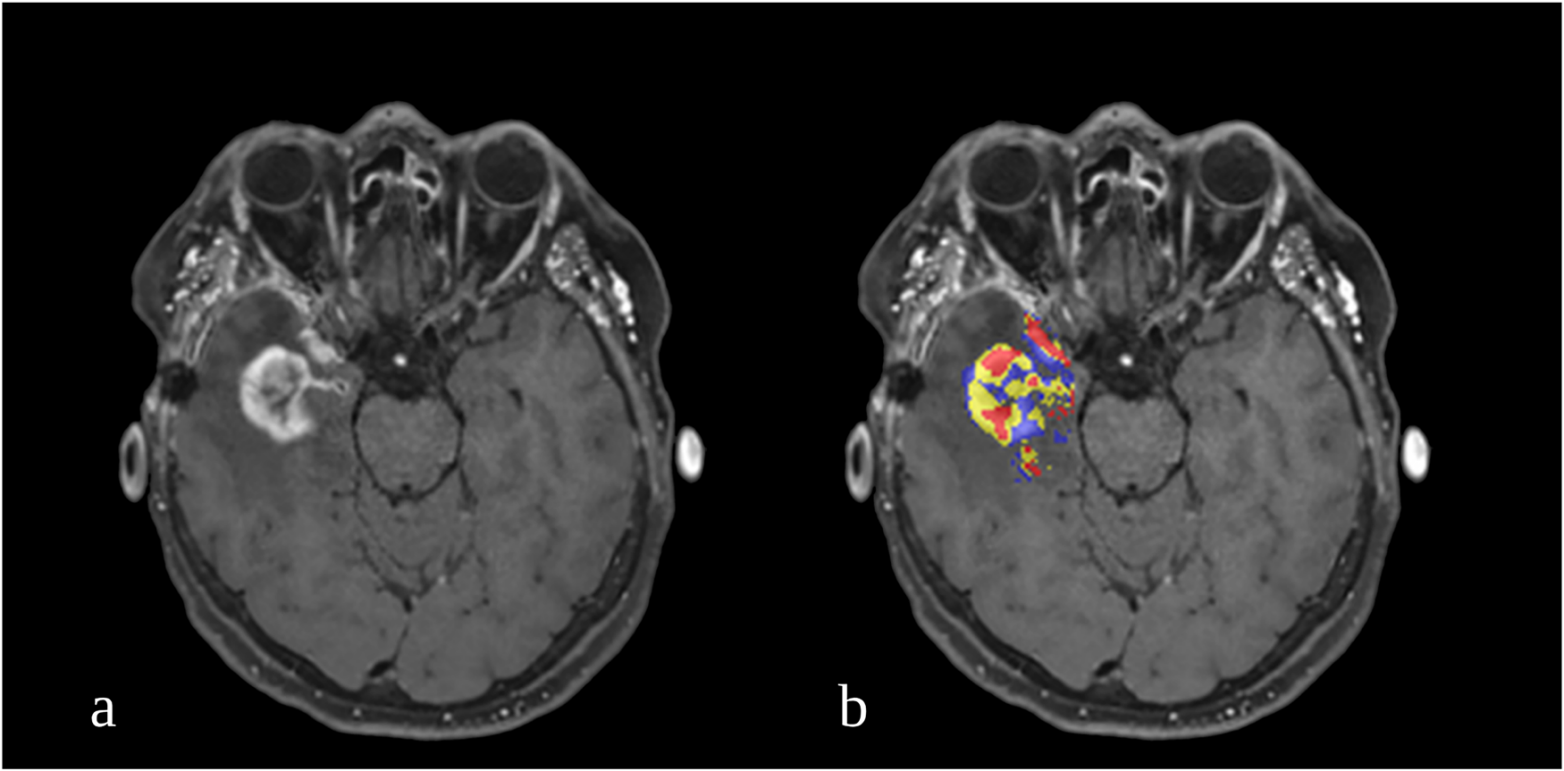
Example of a post-treatment glioblastoma patient with a heterogeneous lesion. An axial T1-weighted post-contrast image (**a**) shows a new contrast-enhancing area in the right temporal lobe. The axial FTB-map overlayed on the T1-weighted image (**b**) shows the heterogeneous aspect of the lesion, indicating that the voxels which were classified as FTB_high_ (red) were mostly found in the lateral and frontal aspects of the lesion, whereas the mediocaudal part of the lesion predominantly contains voxels classified as FTB_mid_ (yellow) and FTB_low_ (blue). FTB, Fractional tumor burden

Quantitative postprocessing of DSC data to construct fractional tumor burden (FTB) maps has been demonstrated to yield a high diagnostic accuracy for the early detection of TP in glioblastoma patients [14]. The FTB is a perfusion-derived metric and is defined as the volume fraction of voxels within a volume of interest (VOI), which have a value above specified standardized relative cerebral blood volume (rCBV) thresholds that are known to correlate with tumor tissue. Thus, FTB maps are considered capable of reflecting glioblastoma lesion heterogeneity as they account for the entire T1 contrast-enhancing lesion [14]. A clear example of the lesion heterogeneity visualized by an FTB-map can be seen in Fig. 1.

However, only limited evidence is available on the use of FTB maps in post-treatment radiological surveillance of glioblastoma patients. Therefore, this study investigated the diagnostic accuracy of DSC-derived FTB quantification in a database of post-treatment glioblastoma patients to distinguish TP from TRA.

## Materials and methods

### Ethical approval

The need for ethical approval was waived by our local ethics committee (Radboudumc, METC Oost-Nederland) due to the retrospective, noninvasive design of this study and the usage of anonymized data. The results of this study did not alter the standard of care of glioblastoma patients, and the methods did not include any additional imaging sessions and/or sequences. Patients who opted out of anonymous data sharing for scientific or educational purposes were excluded.

### Patient inclusion

Adult glioblastoma patients with suspected recurrence after surgical resection, chemotherapy, and irradiation were included retrospectively if they did not opt out of participation in research studies. This was done between July 2021 and July 2022. Inclusion criteria comprised: (i) 18 years of age or older with glioblastoma (based on criteria from World Health Organization 2021 [15]) initially treated with a surgical resection followed by chemo- and radiotherapy following the Stupp protocol [16]; (ii) enlarging or new contrast-enhancing mass on follow-up MRI; (iii) availability of DSC-MRI sequences; (iv) either surgical resection/biopsy of the suspicious lesion or clinical and radiological follow-up with final diagnosis made during multidisciplinary team meetings. Exclusion criteria comprised: (i) only non-enhancing lesions, hyperintense on T2-weighted fluid-attenuated inversion-recovery (FLAIR) images; (ii) susceptibility artefacts related to post-operative hemorrhage; and (iii) imaging artefacts caused by surgical materials. Clinical demographics, molecular information on the tumor, and treatment history were extracted from the electronic patient report. MRI data were extracted from the picture archiving and communication system. Final diagnosis was made based on radiological follow-up using the Response Assessment in Neuro-Oncology (RANO) criteria [2] at least three months after the suspicious lesion was noted on imaging, after which the diagnosis was discussed in a multidisciplinary team meeting. Alternatively, the final diagnosis was made based on histopathological data of the lesion.

### MRI acquisition protocol

MRI examinations were obtained using a 1.5-T scanner (MAGNETOM Avanto Fit, Siemens Healthineers). The clinical protocol included, among other sequences, a DSC sequence acquired using a gradient-echo echo-planar imaging sequence during contrast administration with a first bolus of gadoterate meglumine (Dotarem, Guerbet) 0.1 mL/kg bodyweight at a flow rate of 5 mL/s, followed by a second bolus of 20 mL at a flow rate of 5 mL/s. The sequence technical parameters were as follows: repetition time 1,350 ms; echo time 43 ms; flip angle 60°; voxel size 1.8 × 1.8 × 5.0 mm^3^; acquisition time 2 min; 15 axial sections through the brain, 75 dynamic frames. Furthermore, the protocol included a T1-weighted magnetization‑prepared rapid acquisition gradient-echo‒MPRAGE sequence (repetition time 2,100 ms; echo time 2.42 ms; isotropic voxel size 1 mm^3^; inversion time 900 ms; acquisition time 5 min) and a contrast-enhanced T1-weighted three-dimensional turbo spin-echo sequence (repetition time 600 ms; echo time 7.1 ms; isotropic voxel size 1 mm; acquisition time 3:26 min:s).

### Image processing and quantitative analysis

OsiriX MD (version 3.0, http://www.osirix-viewer.com) and a commercially available plugin, IB Rad Tech (Imaging Biometrics), were used for image processing and the construction of the FTB maps. IB Rad Tech was equipped with a semiautomatic lesion segmentation tool and a leakage-correction algorithm and can be used to process DSC data and calculate the rCBV values per voxel within a defined VOI.

The incorporated leakage-correction algorithm, as described by Boxerman et al [17], uses linear fitting to normalize the rCBV based on the contralateral hemisphere, removing T1 contamination caused by contrast extravasation. In more recent years, modifications to this algorithm have been published, yet these adaptations have yet to be thoroughly evaluated and, as such, have not yet been implemented in commercially available software [18]. The IB Rad Tech workflow generates a ΔT1-weighted image in which the intensity changes of the unenhanced and contrast-enhanced T1-weighted images were detected and quantified.

First, the volume of the semi-automatic segmented VOI of contrast enhancement was determined. This process was conducted by two student-researchers (R.d.W. and B.S.), after which a more experienced researcher (D.H., 7 years of experience with experimental neuroimaging) assessed all segmentations. All researchers involved in the post-processing were blinded to the diagnosis. Segmentations were qualified as incorrect or unsuitable when: (i) the data within the VOI suffered from susceptibility artefacts (*e.g*., hemorrhages); (ii) when the VOI covered parts of non-pathological brain structures (*e.g*., meningeal membranes, vasculature structures, ventricles); or (iii) when the VOI did not cover the entire contrast-enhancing lesion. If necessary, this researcher corrected the segmentations.

Second, co-registration of the rCBV map and the post-contrast T1-weighted images was conducted automatically. Standardization of the rCBV values was performed using the protocol developed by Bedekar et al [19].

Then, the VOI was transferred to the rCBV map, which yielded standardized rCBV perfusion metrics for each voxel within the VOI. The output of the workflow of IB Rad Tech concerned the rCBV maps, FTB maps, and histograms in which all voxels of the VOI were classified into the respective FTB classes. To categorize the voxels within the VOI, thresholds of standardized rCBV values were defined as ratios of 1.0 [20–22] and 1.6 [22, 23]. These values were chosen as literature suggests they are able to differentiate between TP and TRA. Thereby, three FTB classes were created: FTB_low_ (rCBV ≤ 1.0); FTB_mid_ (rCBV between 1.0 and 1.6), and FTB_high_ (rCBV ≥ 1.6). FTB maps and histograms of the FTB class distribution within the VOIs were generated for each patient.

**Fig. 2:**
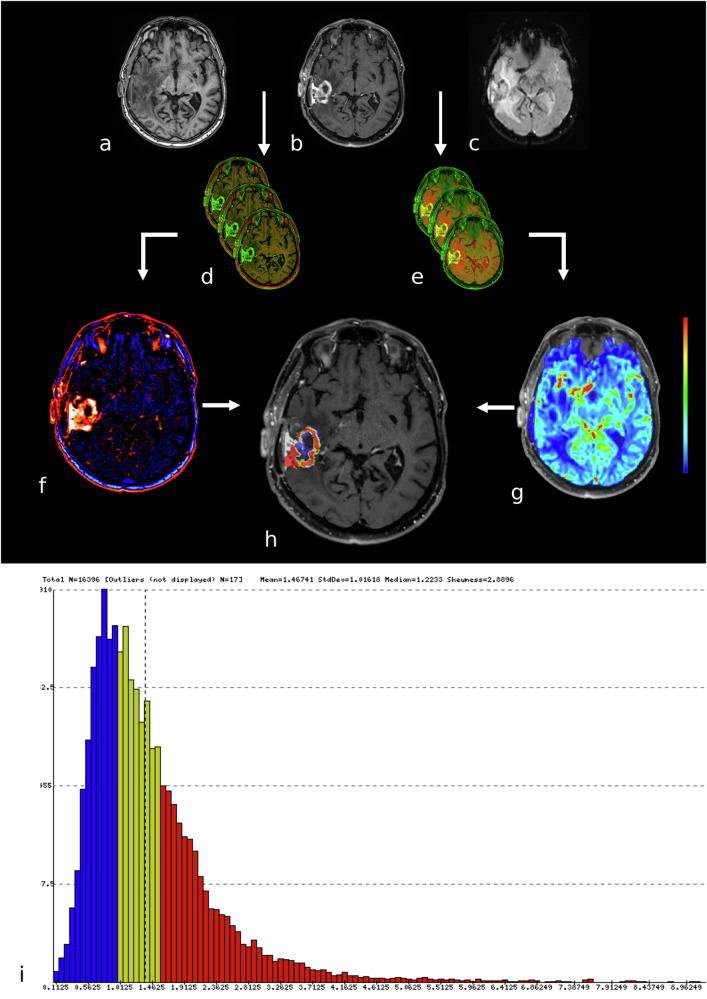
Pipeline of FTB quantification methodology. Axial T1-weighted MRI before (**a**) and after contrast administration (**b**), as well as perfusion-weighted images at the same level (**c**), of a male patient with glioblastomas. The DSC-perfusion weighted data were used as input sequences. T1-weighted unenhanced and contrast-enhanced images were automatically co-registered (**d**) after which the ΔT1 map was created (**f**) by subtracting the unenhanced image from the post-contrast image. Thereby, the regions of contrast-enhancement are clearly visible. The ΔT1 map was used to outline the region of interest on different slides. The T1-weighted post-contrast axial data and the DSC-perfusion weighted data were co-registered as well (**e**). This resulted in an overlay of DSC-data on the T1-weighted post-contrast images (**g**). The VOI and the overlay of DSC-data on the T1-weighted post-contrast images were combined to create the FTB map (**h**) and histogram (**i**). Red represents FTB_high_, yellow indicates FTB_Mid_, and blue indicates FTB_low_. Note that dural thickening was considered a normal post-operative phenomenon by the radiologist assessing this specific patient. DSC, Dynamic susceptibility contrast; FTB, Fractional tumor burden

The pipeline described here is visualized in Fig. 2.

### Statistical analysis

SPSS (Version 27; IBM SPSS Statistics) was used to perform the following analyses. Descriptive statistics were done to report patient demographics and perfusion metrics. The mean FTB coverage per group was calculated by averaging the VOI coverage percentages of all patients within the respective group. A nonparametric Mann-Whitney *U* test was used to compare FTB quantification between the TP and TRA groups. The performance of each significantly different group of FTB classes to distinguish TP from TRA was evaluated with the area under the receiver operating characteristic curve (AUROC). R (version 4.3.1, R Foundation for Statistical Computing) was used to perform bootstrap resampling to internally validate the data used to form the ROC curve. The AUROC was used as a measure of diagnostic accuracy (AUROC 0.5‒0.6 = unsatisfactory, 0.6‒0.7 = fair, 0.7‒0.8 = good, 0.8‒0.9 = very good, and > 0.9 = excellent [24, 25]). The Youden index was used to determine the cutoff values that yielded the optimal sensitivity and specificity. A *p*-value ≤ 0.050 was considered statistically significant for all analyses. Finally, a bootstrap analysis was used to compare the performance of the created AUROC curves, as provided in the pROC package for R [26, 27]. As this was a pilot study, no pre-hoc power analysis was performed.

## Results

In total, 59 patients (32 males) aged 58.0 ± 11.6 years (mean ± standard deviation) with post-treatment glioblastoma who were suspected of tumor recurrence (TP) were included. An overview of the patients included is provided in Table 1. There were no significant differences in age, sex, or MGMT promoter hypermethylation between the TP and TRA groups. Of the included patients, 35 (59%) turned out to suffer from TP during follow-up. For 9 patients, the diagnosis was based on histopathological biopsy; in all other cases diagnosis was made based on clinical and radiological follow-up. The median interval between index MRI and diagnosis was 18 months.

**Table 1:** Characteristics of the included post-treatment glioblastoma patients

Patient characteristics	Overall (*n* = 59)	TP (*n* = 35)	TRA (*n* = 24)
Age	58.0 ± 11.6	55.0 ± 12.2	62.4 ± 9.1
Sex	32 males, 27 females	16 males, 19 females	16 males, 8 females
MGMT promoter hypermethylation	*n* = 39	*n* = 21	*n* = 18
Mean percentage of VOI showing FTB_low_/FTB_mid_/FTB_high_	50%/19%/31%	44%/19%/37%	60%/19%/21%

*FTB* Fractional tumor burden, *MGMT* O^6^‑methylguanine‑deosxyribonucleic acid methyltransferase

The mean percentages of voxels classified as FTB_low_ and FTB_high_ were significantly different between the two groups (*p* = 0.031 and *p* = 0.010, respectively). FTB_low_ turned out to be higher in the TRA group (TRA 60.3%, TP 43.6%), while FTB_high_ was higher in the TP group (TRA 21.2%, TP 37.1%). The percentages of voxels classified as FTB_mid_ were not significantly different between the two groups (*p* = 0.061).

**Fig. 3:**
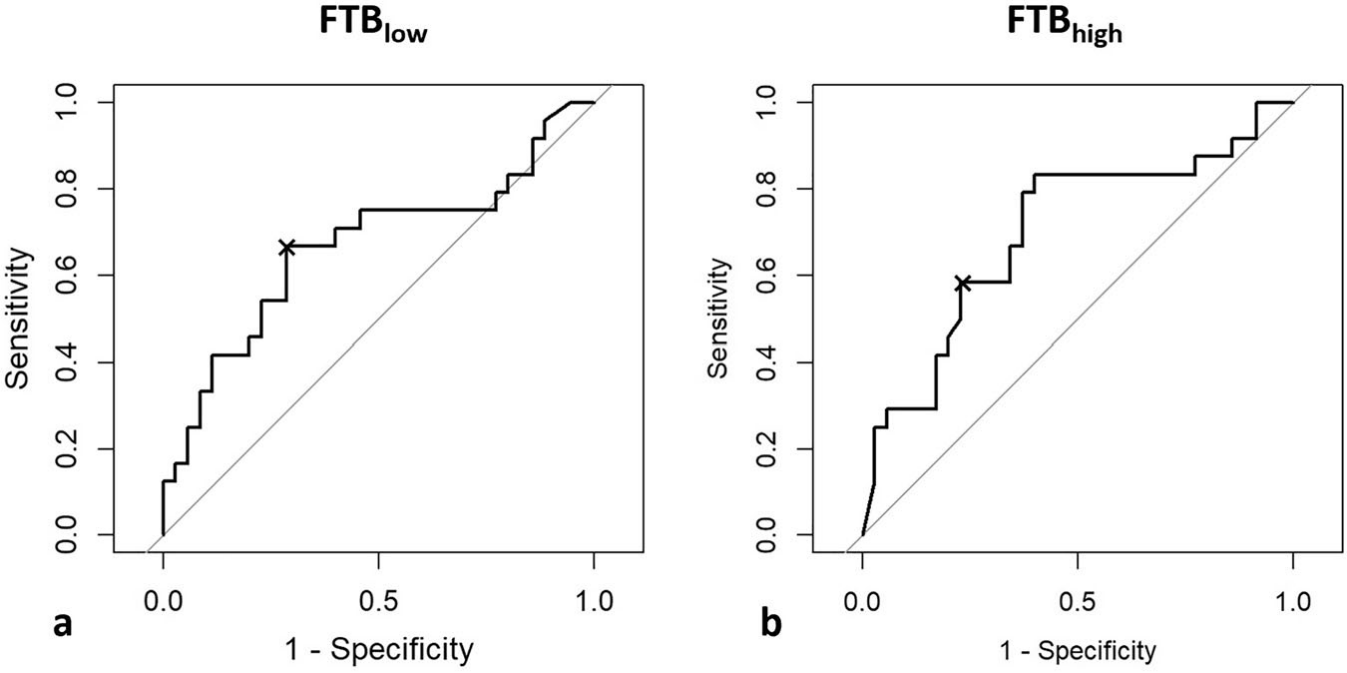
ROC analysis of FTB_low_ and FTB_high_ values regarding the differentiation between TP and TRA. **a** ROC curve of the diagnostic accuracy of the FTB_low_ percentages shows an AUROC of 0.67 (95% confidence interval: 0.52‒0.82). An optimal cutoff value of 59% yielded a sensitivity and specificity of 67% and 71%. **b** ROC curve of the diagnostic accuracy of the FTB_high_ percentages shows an AUROC of 0.70 (95% confidence interval: 0.56‒0.84). Using the cutoff value of 24%, sensitivity and specificity of 63% and 79% were obtained. AUROC, Area under the ROC curve; FTB, Fractional tumor burden; ROC, Receiving operating characteristics

When the percentage of voxels classified as FTB_low_ was used as a diagnostic test to diagnose TRA, an AUROC of 0.67 (95% confidence interval: 0.52‒0.82) was yielded (Fig. 3a). An optimal cutoff value of 59% was determined, indicating that when more than 59% voxels were classified as FTB_low_, the lesion is most likely a TRA lesion. Using this cutoff value, a sensitivity and specificity of 67% and 71% was obtained.

When the percentage of voxels classified as FTB_high_ was used as a test for early detection of TP, the AUROC was 0.70 (95% confidence interval: 0.56‒0.84) (Fig. 3b). An optimal cutoff value of 24% was determined, indicating that when more than 24% voxels were classified as FTB_high_, the lesion would most probably concern a TP lesion. Using this cutoff value, a sensitivity and specificity of 63% and 79% was obtained.

Comparing the two AUROC’s with a bootstrap analysis resulted in a non-significant difference (*D* = -0.729, boot.*n* = 10,000, *p* = 0.466).

## Discussion

The present study shows that the use of two-threshold FTB quantification can distinguish TP from TRA in post-treatment glioblastoma patients with reasonable accuracy. Furthermore, we found no significant difference in MGMT promoter hypermethylation status between the TP and TRA groups, where we expected to see more hypermethylation in the TRA group. The lack of this difference emphasizes the multifactorial influences that are involved in TRA, meaning MGMT methylation status is not a definitive marker. Conclusions based on this MGMT hypermethylation cannot be made based on this study, due to the limited sample size, especially in the TRA group. Our findings corroborate the results of previous authors who showed that the use of the same two rCBV thresholds to define low and high FTB allows for the differentiation of TP from TRA. In their study, retrospective analysis of 47 patients with glioblastoma, astrocytoma grade 3, or gliosarcoma showed an AUROC of 0.88, 0.63, and 0.81 for the FTB_low_, FTB_mid_, and FTB_high_ groups, respectively. For the early detection of TP, the FTB_high_ cutoff point of > 24% yielded a sensitivity and specificity of 80% and 82%, respectively [14]. Qualitative assessment of the FTB images in the same study showed fair interobserver agreement with an intraclass correlation coefficient of 0.48. This might suggest that, despite automated processing of the images, interpretation in routine clinical practice remains challenging [14]. Furthermore, as described in Hu et al, the post-processing methods used should be taken into account when using set thresholds, as reported thresholds can be specific to the used post-processing method.

Compared to other advanced imaging techniques, the reported performance metrics of FTB-maps are mediocre. For example, F-FET (O-(2- [^18^F]fluoroethyl)-l-tyrosine) amino-acid positron emission tomography has been shown to reach a sensitivity of up to 99% and a specificity of 94% [28]. Magnetic resonance spectroscopy has been reported to reach a sensitivity and specificity of 91% and 84%, respectively [29]. While chemical exchange saturation transfer MRI has been reported to reach a sensitivity and specificity of 85% and 88%, respectively [30]. These techniques are, however, limited to large academic centers, requiring technical expertise. In order for these techniques to aid on a larger scale, a widespread availability of imaging sequences and standardization methods is required [12]. As DSC perfusion-weighted imaging is already a widely available imaging technique, with commercially available software to create FTB-maps, this technique has a major function to play in the follow-up of glioblastoma patients. Next to the aforementioned developments in advanced MRI, two recent reviews highlighted the potential of two other broader MRI developments in the field of neuro-oncology: the use of deuterium MRI and the use of ultra-high field MRI [31, 32].

Pairing qualitative reading with quantitative reading may increase the diagnostic accuracy of FTB images in clinical practice. As almost all lesions within our cohort showed heterogeneous origin of the contrast-enhancing lesion, the strength of FTB-maps in visualizing heterogeneity could very well impact clinical practice. The heterogeneity within FTB-maps indicates that TP and TRA can occur simultaneously, which is also well known from histopathological studies. The found heterogeneity differed between lesions, which could explain the large amount of variability in the distribution of the FTB classes.

Therefore, qualitative assessment of FTB images in daily radiological practice should be further investigated. The fair interobserver agreement of FTB images as reported by Iv et al seems to be reflected by the previously published interobserver agreement of DSC perfusion data in the post-treatment surveillance of glioblastoma. In a recent study, the semiquantitative assessment of DSC images in 32 post-treatment glioblastoma cases was found to have only moderate interobserver agreement with an intraclass correlation coefficient of 0.63 [33]. This indicates that perfusion-weighted MRI data in general might be difficult to interpret. In a recent review, it was therefore stated that perfusion-weighted MRI data should be evaluated by an experienced reader and should always be interpreted together with other MRI sequences. Furthermore, radiological evolution of the area of interest over time and the clinical context need to be taken into account [34]. In addition, a well-established image-review process needs to be applied upfront to assess perfusion-weighted MRI data, considering the poor repeatability and reproducibility found in a multicenter study using DSC perfusion-weighted imaging [35]. Therefore, the authors recommended rCBV measurement by multiple, experienced readers independently. To resolve disagreements, an adjudicator could be involved to provide the final perfusion measurement [35]. Whether this could help to increase the impact of the qualitative assessment of FTB images remains unknown.

In a previous work, we investigated the performance of a qualitative analysis of FTB maps. This analysis did not provide additional diagnostic accuracy (AUROC_withFTB_ 0.71 *versus* AUROC_withoutFTB_ 0.68) but did increase reader confidence [36]. Furthermore, the durations of FTB-map generation and assessment were recorded; the median times spent were 313 s and 19 s, respectively. A recent study by Yamin et al [37] also illustrates that the inter-rater agreement and consensus were highest when using FTB-maps, compared to other perfusion-based methods. In their study, FTB-based analysis also led to clinically relevant changes in the interpretation of MRI in 2–20% of analyzed cases, depending on the reader.

Recent case reports describe the utilization of FTB-maps in the monitoring of experimental treatment response, with FTB-maps showing promise in predicting treatment response before conventional MRI [38, 39]. While no large cohort studies have been performed, this paves the way for future application of FTB-maps.

Our current findings, combined with recent studies into the potential of FTB-maps, show clear advantages of FTB-map implementation, despite not showing clear additional diagnostic accuracy over conventional DSC-based rCBV analysis: FTB-maps are standardized and comparable between centers and scanner types when using similar post-processing. The FTB-maps provide a clear overview of lesion heterogeneity, allowing the identification of biopsy sites and potentially guidance for future localized treatment options. A complete volume-based analysis, as used in these FTB maps, was also shown to be more accurate than the commonly employed ‘hot spot’ method [40]. Based on these advantages, our recommended clinical implementation of FTB-maps would consist of using both quantitative and qualitative FTB-maps, in combination with other available sequences.

To validate these findings, future research should consist of a prospective, longitudinal study, combining both quantitative and qualitative analysis. Preferably with a larger cohort and in a multi-center setting, to also investigate the impact of standardization on comparability. Furthermore, a prospective study could explore and optimize guidelines for the implementation of FTB-maps in clinical practice. Finally, a prospective study could also assess the evolution of a lesion over time based on FTB parameters, providing insights on regional development of both TP and TRA.

Limitations of the present work concern the relatively limited sample size, the retrospective study design, the usage of single-center data, and the lack of qualitative assessment of the FTB images paired with the quantitative analysis. The limited sample size leads to a lower achieved statistical power, making it more difficult to draw reliable conclusions, especially in interpreting borderline *p*-values, such as the difference in FTB_mid_ voxels between TP and TRA (*p* = 0.061). This emphasizes the need for further prospective studies. The retrospective design and short inclusion period (1 year) increase the chance of misclassification or misattribution of found effects. The usage of single-center data limits the generalizability of our findings; the standardization protocols included in the used software should filter out most inter-scanner variability. The final diagnosis was based on clinical/radiological follow-up, based on the RANO criteria [2] when histopathological assessment was not available. The mixed reference standard and usage of follow-up-based diagnosis increase the chance of incorporation bias, misclassifications, or inaccuracies in diagnosis. However, due to the invasive nature of a stereotactic brain biopsy, it is not part of standard clinical care of post-treatment neuro-oncology patients in the Netherlands. This is why the well-known alternative ‘silver standard’ of clinical and radiological follow-up was chosen, based on a median follow-up time of 18 months. The FTB-data generated for this study did not in any way influence the diagnosis. This approach is deemed unlikely to have a relevant impact on the findings of this study.

The clinical protocol included, among other sequences, a DSC sequence acquired using a gradient-echo echo-planar imaging sequence during contrast administration with a first bolus of gadoterate meglumine (Dotarem) 0.1 mL/kg bodyweight at a flow rate of 5 mL/s, followed by a second bolus of 20 mL at a flow rate of 5 mL/s. This study demonstrated that FTB quantification for the early detection of TP in post-treatment glioblastoma patients yields a fair diagnostic accuracy. However, its potential value in the daily clinical workflow requires further research.

## Data Availability

The datasets generated during and/or analyzed during the current study are available at reasonable request by contacting the corresponding author.

## Abbreviations

AUROC: Area under the receiver operating characteristic curve
DSC: Dynamic susceptibility contrast
FTB: Fractional tumor burden
MGMT: O^6^‑methylguanine‑deosxyribonucleic acid methyltransferase
MRI: Magnetic resonance imaging
rCBV: Relative cerebral blood volume
TP: Tumor progression
TRA: Treatment-related abnormalities
VOI: Volume of interest
